# Use of patient- handling devices and coworker assistance in long-term care settings: A cross-sectional study

**DOI:** 10.1016/j.ijnsa.2025.100317

**Published:** 2025-03-17

**Authors:** Minjung Kyung, Soo-Jeong Lee, Laura M. Wagner, OiSaeng Hong

**Affiliations:** a College of Nursing, The Catholic University of Korea, Seoul, South Korea; b School of Nursing, University of California, San Francisco, San Francisco, California, USA

**Keywords:** Patient-handling devices, Coworker assistance, Musculoskeletal symptoms, Direct care workers, Long-term care

## Abstract

**Background:**

Although many patient handling activities require use of lifting devices and assistance from coworkers to ensure safety and efficiency, integrating these practices into the workplace remains challenging.

**Objective:**

The objectives of this study were to examine the association of musculoskeletal symptoms with the use of patient -handling devices or coworker assistance and to identify factors associated with their adoption among direct care workers in long-term care facilities.

**Methods:**

A cross-sectional study was conducted among 376 direct care workers recruited from 19 long-term care facilities in South Korea. Chi-square tests and analysis of variance with Tukey post-hoc analysis were used to assess differences in musculoskeletal symptom prevalence, frequency, and severity by patient handling methods. Multiple logistic regression was used to examine the relationship between the use of coworker assistance or patient -handling devices and demographic, job-related, physical, and psychosocial work factors and perception of management's safety priority.

**Results:**

The majority of participants were female, married, and employed in non-permanent positions. Among the participants, 42.2% used both patient- handling devices and coworker assistance, 5.8 % used only patient- handling devices, 24.7 % used only coworker assistance, and 27.3 % used neither method. Workers who used both methods reported a significantly lower prevalence, frequency, and severity of musculoskeletal symptoms compared to those who relied on only one method or neither. Older and immigrant workers were less likely to use patient- handling devices. While workers perceiving management having a high priority for safety were more likely to use coworker assistance, immigrant, non-permanent, and overcommitted workers and those with longer years in long-term care showed lower tendencies to seek coworker assistance.

**Conclusion:**

Despite the musculoskeletal benefits of using patient- handling devices and coworker assistance, their availability and adoption remain insufficient in long-term care facilities in Korea. These findings underscore the vital role that nursing leaders may play in promoting the adoption of combined patient handling methods, particularly among vulnerable worker groups. Empowerment strategies, such as fostering a supportive work environment and addressing barriers faced by overcommitted, long-tenured, and marginalized workers, may be essential to improving the safety and well-being of both patients and staff in long-term care settings.

**What is already known**

• Direct care workers in long-term care facilities are subjected to demanding patient handling activities, a primary cause of musculoskeletal disorders.
• Use of patient -handling devices with coworker assistance is an important component of safe patient handling and mobility programs.
• Despite the benefits of patient- handling devices, many workers experience barriers to their use.

Alt-text: Unlabelled box

**What this paper adds**

• The use of patient- handling devices, with or without coworker assistance, was associated with lower prevalence, frequency, and severity of musculoskeletal symptoms among direct care workers in long-term care facilities compared to those who used neither method.
• Older and immigrant workers were less likely to use patient- handling devices.
• Overcommitted workers, those with longer years of experience in long-term care, and marginalized groups, such as older workers, immigrants, and non-permanent workers, were less likely to seek coworker assistance for patient handling.
• Workers who perceived management having a high priority for safety were more likely to utilize coworker assistance.

Alt-text: Unlabelled box

## 1 Introduction

Musculoskeletal disorders remain a major health concern worldwide, despite being largely preventable. In 2020, musculoskeletal disorders were the second leading cause of non-fatal disability globally, and the number of incident cases surged by 50.9 % from 1990 to 2019 (Hanifa et al., 2023; Wu et al., 2021). Musculoskeletal disorders impair mobility and dexterity, leading to reduced work capacity, higher turnover rates, early retirement, and diminished societal participation (Davis & Kotowski, 2015). The situation is particularly dire in long-term care settings, where frail elderly residents heavily rely on physical care and there is a lack of patient-handling devices (Cheung et al., 2018). A study involving registered nurses revealed that the prevalence of work-related musculoskeletal pain was higher across all body regions for those working in long-term care settings compared to their counterparts in hospitals (Lee et al., 2015). Ongoing staff shortages and an aging workforce are expected to exacerbate this issue (Caponecchia et al., 2020; Miller et al., 2023). According to the United States (U.S.) Bureau of Labor Statistics, there were 976,090 cases involving days away from work, job restriction, or transfer due to musculoskeletal disorders in the private sector over 2021–2022 (Bureau of Labor Statistics, 2023). These disorders not only reduce the quality of life for workers but also directly affect the quality of care they can provide.

Direct care workers are trained staff responsible for personal care tasks, such as feeding, bathing, dressing, and toileting (Kim & Tak, 2018). These workers, referred to by various titles across different countries or institutions, include nursing assistants, nursing aides, or personal care aides (Trinkoff et al., 2020). The incidence of musculoskeletal disorders is particularly high among direct care workers due to frequent exposure to ergonomic risk in their daily work routines. In the U.S., nursing assistants report the second-highest number of musculoskeletal disorder cases in the private sector, with a rate of 1.81 lost -time musculoskeletal disorders per 100 full-time equivalents (U.S. Bureau of Labor Statistics, 2020). This rate is significantly higher than the 0.47 per 100 for licensed practical nurses and 0.53 per 100 registered nurses, both of whom are also recognized as high-risk groups for musculoskeletal disorders (U.S. Bureau of Labor Statistics, 2020).

Manual patient handling is a primary cause of musculoskeletal disorders in direct care workers (Davis & Kotowski, 2015; Johnson et al., 2023). An analysis of medical costs associated with workers’ compensation claims for musculoskeletal injuries highlights the significant impact of patient handling. These claims accounted for 34.4 % of total medical costs, representing the largest percentage of all claims in long-term care for one U.S. insurer (Pieretti et al., 2020). To address this, safe patient handling and mobility programs that include a no-lifting policy have been implemented in several countries to encourage safe practices by minimizing manual patient handling and promoting the use of mechanical lifting devices and other assistive technologies. Patient-handling devices —such as mechanical lifts (overhead ceiling lifts, floor-based lifts), hoists, transfer belts and slide sheets, stand-assist devices, and repositioning aids— have demonstrated their potential to decrease biomechanical risk during lifting tasks (Freiberg et al., 2016; Hegewald et al., 2018). A meta-analysis found that the safe patient handling and mobility programs significantly reduce injury risks (Teeple et al., 2017). Specifically, these programs led to a 56 % reduction in the overall risk of injury across all healthcare settings and a 49 % reduction in injury risk within long-term care facilities (Teeple et al., 2017).

Safe patient handling practices often involve teamwork and collaboration with caregivers. The American Nurses Association has established safe patient handling and mobility standards that emphasize the importance of using appropriate equipment and multiple caregivers based on the patient's weight and mobility needs (Mary et al., 2021). For instance, these standards recommend that patients weighing over 200 pounds may require at least two caregivers, and, in some cases, three to four caregivers when using equipment such as ceiling lifts or air-assisted lateral transfer devices (Mary et al., 2021). The U.S. Occupational Safety and Health Administration has specified recommendations on the number of caregivers required when using patient handling devices to protect workers from musculoskeletal injuries (OSHA, 2009). It also suggests using a lateral sliding aid and three caregivers or a friction-reducing device with two caregivers for patients weighing over 200 pounds (OSHA, 2009).

Integrating safe patient handling and mobility guidelines into daily routines presents challenges for many direct care workers. Despite the recognized safety benefits of patient-handling devices, their use remains limited worldwide (Kucera et al, 2019, Myers et al., 2012). The situation is particularly concerning in South Korea. Although the Korea Occupational Safety and Health Administration promotes the use of patient handling devices, most hospital and long-term care facilities lack these devices, and workers rarely rely on coworker assistance for patient handling (Korea Occupational Safety and Health Administration, 2012). Kim and Song (2023) revealed that while six out of 10 workers are highly aware of the necessity of patient handling devices, only one out of 10 direct care workers actually used them in clinical practice. This discrepancy highlights the gap between awareness and implementation. Understanding the key components of safe patient handling and mobility practices, including the use of patient-handling devices and coworker assistance, is essential for preventing musculoskeletal disorders among direct care workers. However, researchers predominantly have focused on the prevalence of musculoskeletal disorders and the risks of manual patient handling, offering little insight into actual usage trends. To date, no \researchers have accurately examined how many workers in South Korea use patient handling devices and coworker assistance in practice. Investigating these trends is critical to addressing gaps in safe patient handling and mobility implementation. Therefore, we aimed to (1) examine the association of musculoskeletal symptoms with the use of patient-handling devices or coworker assistance; and (2) identify factors associated with the use of patient-handling devices and coworker assistance among direct care workers in long-term care facilities in South Korea.

## 2 Methods

### 2.1 Study design, sample, and data collection

In this cross-sectional study, we included a convenience sample of 377 direct care workers in long-term care facilities in South Korea. Long-term care facilities were recruited from three cities in Gyeonggi Province, which represents 26 % of the Korean population (Korean Statistical Information Service, 2022b). Direct care workers employed in their current job for at least 3 months, with the ability to read, write, and understand Korean, were included in the study.

The study participants were recruited from May to August 2022, which was during the COVID-19 pandemic when direct access to the facilities was limited. To recruit participants, study information and recruitment letters were distributed via fax or email to 110 long-term care facilities, representing 4.8 % of the 2,267 institutions in the Gyeonggi area (Korean Statistical Information Service, 2022a; Ministry of Health and Welfare of South Korea, 2022). These facilities were selected based on their proximity to the primary investigator. Of 110 facilities contacted, responses were received from 19 long-term care facilities, which granted permission to post a flyer with contact information on their department bulletin boards; the bed capacities ranged from 9 to 196.

Data were collected with a self-administered questionnaire, after testing and validating its content and structure through a pilot study with 20 direct care workers in a long-term care facility. The survey was distributed and collected during a single monthly staff meeting or during the required training programs offered by the Korea National Health Insurance Service. As an incentive, a 10,000 Korean won (US $8) gift was provided to each participant upon survey completion. A power analysis using the G*Power 3.1.9.7 program (Universität Düsseldorf, Düsseldorf, Germany) indicated that 280 participants would be required to achieve a medium effect size of 0.25, with a significance level of 0.05 and a power of 0.95 for an F-test to compare differences across four groups. A total of 433 direct care workers expressed interest and received the survey, of whom 403 completed it, resulting in a response rate of 93 %. Of these, 25 participants were excluded due to either less than 3 months of direct care worker experience (*n* = 11) or missing responses of 5 % or more (*n* = 15). The analysis included a final sample of 377 participants. Ethical approval for the study was obtained from both the Committee on Human Research of the University of California, San Francisco in the United States and the Public Institutional Review Board in South Korea.

### 2.2 Measures

**Dependent variables** were the use of coworker assistance and patient handling devices for patient handling tasks. Respondents were asked two separate questions regarding their practices over the past 12 months: whether they ever included coworkers when performing patient handling tasks and if they ever utilized any patient handling devices. Given the very limited availability of patient lifting equipment in Korea, we considered patient handling devices to include mechanical lifts (overhead ceiling lifts, floor-based lifts), hoists, transfer belts and slide sheets, stand-assist devices, and repositioning aids. The outcome variable was initially categorized into four groups: (1) using both patient handling devices and coworker assistance, (2) using only patient handling devices, (3) using only coworker assistance, and (4) using neither.

**Independent variables** were demographics, job characteristics, physical work factors, and psychosocial work factors, perception of management's safety priority, and musculoskeletal symptoms (Kucera et al., 2019; Lee et al., 2010). **Demographic variables** included age, sex, immigrant status, marital status, and education level. **Job characteristics** included years worked in long-term care and work arrangement. Work arrangement was categorized into two groups: permanent workers and non-permanent workers, which included temporary workers and independent workers. Unlike temporary workers employed by a contracting company, independent workers are self-employed individuals who provide services to clients under specific terms (Krueger, 2018). In South Korea, a significant portion of direct care workers in long-term care hospitals are classified as non-permanent workers. These workers are often hired through outsourcing agencies or directly by patients, resulting in a multi-party employment relationship characterized by temporary and independent work statuses (Kim et al., 2016; Kwon et al., 2022).

**Physical work factors** were measured by a single question asking respondents to rate their level of perception about how strenuous their work was on a 5-point Likert scale (1 ‘not strenuous’, 5 ‘extremely strenuous’)(Neupane et al., 2020).

**Psychosocial work factors** were measured using the Korean version of the Effort-Reward Imbalance questionnaire (Eum et al., 2007). This questionnaire consists of three components: effort (six items), reward (10 items), and overcommitment (six items) and has been validated in various studies (Eum et al., 2007; Hwang et al., 2018; Siegrist, 1996; Siegrist et al., 2004). Effort represents job demand or obligations (e.g., “I have constant time pressure due to a heavy workload”), reward encompasses what workers can gain from their work (e.g., “I receive the respect I deserve from my superior or a respective relevant person”), and overcommitment reflects a cognitive-motivational pattern that leads workers to exert excessive effort in their job, being unable to detach from work (e.g., “I get easily overwhelmed by time pressures at work”) (Siegrist et al., 2004). Based on the pilot test results, we revised one item of the Effort-Reward Imbalance Questionnaire from 'My job promotion prospects are poor' to 'The likelihood of my job promotion is low' to enhance internal consistency. All scale items used a 4-point Likert scale (1 ‘strongly disagree’, 4 ‘strongly agree’). Effort, reward, and overcommitment scores were calculated as the sum of the item responses. The Effort-Reward Imbalance ratio was calculated by dividing effort by rewards and applying a correction factor of 3/5. The Effort-Reward Imbalance ratio of 1.0 indicates a balance between effort expended and rewards received (Siegrist et al., 2004).

**Perception of management's safety priority** was defined and adapted from the Nordic Safety Climate Questionnaire. Management's safety priority reflects the degree to which an organization's leadership emphasizes worker safety and well-being as a fundamental operational value (Kines et al., 2011). For simplicity and efficiency, management's safety priority in this study was assessed by a single question: “Do you think the health and safety of workers are a high priority where you work?” (Kines et al., 2011). A ‘yes’ response indicated a high management's safety priority, while a ‘no’ indicated a low management's safety priority.

**Musculoskeletal symptoms** were assessed for presence, frequency, and severity using a modified version of the questionnaire from the Nurses’ Work Life and Health Study (Lipscomb et al., 2002). The modified questionnaire adhered to the definition of musculoskeletal symptoms as outlined in the Nordic Musculoskeletal Questionnaire (Kakaraparthi et al., 2023). Symptoms assessed included pain, aching, stiffness, burning, numbness, or tingling in various body regions, such as upper extremities, neck and shoulder, back, and lower extremities. Respondents were asked if they experienced any work-aggravated or work-caused musculoskeletal symptoms within the past 12 months. The timeframe was chosen to reflect both recent and cumulative exposures while minimizing recall bias. While this timeframe allowed us to assess the prevalence of symptoms indicative of longer-term occupational risks, it also encompassed acute incidents, as symptoms could arise from either single events or cumulative exposures over time. The frequency of these symptoms was assessed using a 6-point Likert-type scale (1 ‘never’, 6 ‘daily’), and their severity was assessed using a 5-point Likert-type scale (1 ’none’, 5 ‘extreme’).

### 2.3 Data analysis

The study variables were summarized using descriptive statistics, including frequency, percentage, mean, and standard deviation. Chi-square tests were conducted to compare differences in the prevalence of musculoskeletal symptoms based on the use of coworker assistance and patient-handling devices, while analysis of variance (ANOVA) was employed to assess differences in symptom frequency and severity. To further explore group differences, Tukey post-hoc analysis was performed. For the multivariable analysis, we focused on workers who had access to patient-handling devices in their workplace. This approach ensured that the analysis captured factors influencing individual behavioral intention. Due to the small sample size in each group categorized by patient-handling device availability, factors associated with the use of patient handling devices and coworker assistance were analyzed separately. Given the binary nature of the outcome variables, multivariable logistic regression analyses were conducted. Based on initial screening and clinical relevance, variables with *p*-values less than 0.05 in the bivariate analysis were included in the regression model. Sex and marital status were excluded due to highly disproportionate distributions, and the effort variable was removed after addressing multicollinearity concerns. Additionally, management's safety priority was excluded from the analysis of patient handling device use due to an extremely wide confidence interval (CI), which made the estimates unreliable. Odds ratios (OR) and 95 % CIs were reported to provide a comprehensive interpretation of the associations between independent variables and the likelihood of using patient handling methods. All data analyses were performed using STATA version 16.0 (Stata Corporation, College Station, TX).

## 3 Results

### 3.1 Characteristics of study participants

Table 1 summarizes the characteristics of 376 study participants. The sample predominantly comprised women, with nearly one third identifying as immigrants. Most participants were married and non-immigrant and had a high school education or less. The average age was around 60 years, with a mean employment duration of about 6 years as direct care workers. The majority were permanent employees, and more than half reported their management prioritized worker safety.

**Table 1:** Sample characteristics of direct care workers in long-term care settings.

Characteristics	Total (*N* = 376)
	*N* (%)
Sex (female)	326 (86.9)
Immigrant status (immigrant)	104 (27.8)
Marital status (Married)	353 (95.9)
Education	
Less than middle school graduate	68 (18.2)
High school graduate	256 (68.7)
College 1 year or more	49 (13.1)
Non-permanent workers	269 (72.0)
Management safety priority (high)	210 (55.8)
	Mean (SD)
Age, years	60.7 (6.4)
Years worked in long-term care	5.9 (4.9)
Perceived exertion (1-5)	3.7 (0.7)
Effort (6-24)	14.3 (2.6)
Reward (10-40)	269 (3.5)
Overcommitment (6-24)	13.5 (2.4)
Effort-reward imbalance (0.25-4)	0.91 (0.25)

Note. Sample sizes for variables differ due to missing data.

Abbreviation: N, the total number of participants in a given group; SD, Standard Deviation.

Temporary workers or independent workers.

**Fig. 1:**
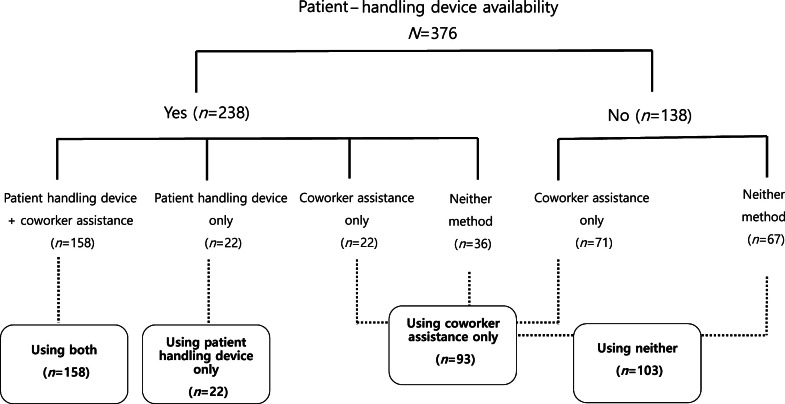
Distribution of patient handling methods based on device availability among direct care workers. Abbreviation: N, the total number of participants in a study; n, the number of participants in a specific group.

Fig. 1 illustrates the distribution of patient-handling methods based on the availability of patient-handling devices among the 376 participants. More than 60 % of the participants reported having access to patient- handling devices. Among those with device availability, two thirds used both patient -handling devices and coworker assistance, while the remainder used only patient-handling devices, only coworker assistance, or neither method in the past 12 months. Among those without device availability, half did not use any patient handling methods. Overall, 42.0 % of participants reported ever using both patient handling devices and coworker assistance, 5.9 % reported ever using only patient handling devices, 24.7 % reported ever using only coworker assistance, and 27.4 % reported never using any method in the past 12 months.

### 3.2 Musculoskeletal symptoms

Table 2 shows the prevalence, frequency, and severity of musculoskeletal symptoms among participants based on their use of coworker assistance and patient handling devices. Workers who used both methods reported the lowest prevalence of musculoskeletal symptoms, while those who used neither method had the highest. In the post-hoc analysis, the prevalence of musculoskeletal symptoms differed significantly across all groups, except between those using both methods and only patient handling devices, and between those using only coworker assistance and neither method. Symptom frequency and severity were also lowest among workers using both methods. The frequency of symptoms differed significantly across all groups, except between those using both methods and only patient handling devices and between those using only coworker assistance and neither method. For symptom severity, the only non-significant difference was between workers using both methods and only patient-handling devices.

**Table 2:** Musculoskeletal symptoms by use of patient handling methods.

	Patient handling devices + coworker assistance^a^ (*n* = 158)	Patient handling devices only^b^ (*n* = 22)	Coworker assistance only^c^ (*n* = 93)	Neither patient handling devices nor coworker assistance^d^ (*n* = 103)	*p* *Post-hoc*
	*N* (%)	*N* (%)	*N* (%)	*N* (%)	
Musculoskeletal symptoms*	70 (45.4)	14 (60.6)	74 (80.4)	81 (81.8)	**<.001** a<c a<d b<c b<d
	Mean (SD)	Mean (SD)	Mean (SD)	Mean (SD)	
Symptom frequency (1-6)	2.63 (1.86)	2.66 (1.86)	4.29 (1.80)	4.29 (1.89)	**<.001** a<c a<d b<c b<d
Symptom severity (1-5)	2.27 (1.12)	2.34 (0.88)	3.15 (1.02)	3.59 (1.17)	**<.001** a<c a<d b<c b<d c<d

⁎ Experiencing musculoskeletal symptoms in the past 12 months Abbreviation: N, the total number of participants in a given group; n, the number of participants in a specific group; p, *p*-values indicate statistical significance from ANOVA; SD, Standard Deviation.

### 3.3 Factors associated with using patient handling devices and coworker assistance

Table 3 presents the factors associated with the utilization of patient-handling devices and coworker assistance among direct care workers. In the bivariate analysis, age, immigrant status, work arrangement, job tenure, management's safety priority, reward, and overcommitment were associated with the use of patient handling devices. Multivariable analysis indicated that older workers and immigrant workers were significantly less likely to adopt patient handling devices.

**Table 3:** Factors associated with the use of patient handling methods: bivariate and multivariable logistic regression analysis.

	Patient handling device use (*n* = 238)				Coworker assistance use (*n* = 376)			
	OR	95 % CIs	OR	95 % CIs	OR	95 % CIs	OR	95 % CIs
Age	**0.91**	**0.87 – 0.96**	**0.92**	**0.86 – 0.98**	**0.91**	**0.88 – 0.95**	0.98	0.93 – 1.03
Immigrant	**0.16**	**0.08 - 0.31**	**0.20**	**0.09 -0.42**	**0.08**	**0.05 – 0.14**	**0.09**	**0.04 – 0.17**
Education (ref. Less than middle school graduate)								
High school graduate	1.28	0.58 – 2.81			1.38	0.78 – 2.41		
College 1 year or more	0.91	0.33 – 2.51			0.61	0.29 – 1.28		
Non-permanent workers	**0.31**	**0.14 – 0.69**	0.56	0.23 – 1.37	**0.31**	**0.18 – 0.55**	**0.46**	**0.22 - 0.98**
Years worked in long-term care	**0.93**	**0.87 – 0.99**	1.02	0.94 – 1.12	**0.86**	**0.82 – 0.9**	**0.87**	**0.82 – 0.93**
Perceived exertion	1.05	0.68 – 1.63			0.84	0.62 – 1.13		
Management safety priority (high)	**37.93**	**16.67 – 91.85**			**3.37**	**2.15 – 5.28**	**3.60**	**1.88 – 6.91**
Reward	**1.17**	**1.07 – 1.29**	1.12	0.99 – 1.26	**1.14**	**1.07 – 1.22**	1.07	0.98 – 1.18
Overcommitment	**0.86**	**0.75 – 0.99**	1.01	0.86 – 1.2	**0.79**	**0.71 – 0.87**	**0.87**	**0.76 – 0.99**
Effort-reward imbalance	0.66	0.16 – 2.66			0.71	0.29 – 1.71		

Abbreviation: n, the total number of participants in a given group; OR, odds ratio; 95 % CIs, 95 % Confidence Intervals.

For the use of coworker assistance, similar factors—age, immigrant status, work arrangement, job tenure, management's safety priority, reward, and overcommitment—were significant in the bivariate analysis. In the multivariable analysis, immigrants, non-permanent workers, overcommitted workers, and those with longer job tenure in long-term care were less likely to use coworker assistance for patient handling. Conversely, workers who perceived high management's safety priority were more likely to seek coworker assistance compared to those with lower perceptions.

## 4 Discussion

We investigated factors associated with using patient-handling devices and coworker assistance and their relationship with the experience of musculoskeletal symptoms among direct care workers in long-term care facilities in South Korea. While repositioning aids were included as patient-handling devices, many facilities lacked access to these tools, and a significant number of workers did not use any safe handling methods. Using patient-handling devices, either alone or with coworker assistance, showed significant inverse associations with musculoskeletal symptoms, suggesting their protective effects. However, from our findings, we have indicated that workers with overcommitment, longer job tenure in long-term care, and those in marginalized groups, such as older workers, immigrants, and non-permanent workers, were less likely to adopt these methods. Conversely, a high management's safety priority was associated with an increased likelihood of using coworker assistance.

### 4.1 Musculoskeletal symptoms and the use of patient-handling devices and coworker assistance

Researchers have presented evidence that highlights the benefits of patient-handling devices in reducing musculoskeletal disorders (Aljohani & Pascua, 2019; Li, 2004). Lee et al. (2013) reported that work-related shoulder and low back symptoms were less common among nurses equipped with lifts than among nurses without access to lifts. Trinkoff et al. (2001) also noted a protective effect of lifting devices on lower back pain among nurses. From our findings, we have contributed to this body of evidence. Workers who used neither patient-handling devices nor coworker assistance were significantly more likely to experience work-related musculoskeletal symptoms, which were also more frequent and severe, compared to those using both methods. On the other hand, the use of patient-handling devices—whether with or without coworker assistance—was significantly associated with lower prevalence, frequency, and severity of musculoskeletal symptoms. These findings suggest that the protective effect is primarily driven by patient-handling devices rather than coworker assistance, aligning with biomechanical research (Richarz et al., 2023). Interestingly, symptom severity was also lower among workers who relied only on coworker assistance than those who used neither method. Although manual patient handling is widely regarded as hazardous (Marras et al., 1999)—whether conducted by one or two handlers—it remains common and often unavoidable in clinical and care settings. This challenge is particularly pronounced in Korea, where mechanical lifts are rarely available. Even in facilities with such devices, environmental constraints often limit their use. Consequently, many care workers are frequently exposed to manual handling tasks. We suggest that coworker assistance, while not fully mitigating the risk of injury, offers better protection for musculoskeletal health compared to one-person manual handling when patient handling devices are unavailable and manual handling is inevitable.

### 4.2 Factors associated with the use of patient handling devices and coworker assistance

We identified several factors associated with the use of patient-handling devices and coworker assistance. Among demographic characteristics, age and immigrant status emerged as significant factors of utilization. Older workers were less likely to use patient handling devices, which may be attributed to challenges in adopting new technologies. This is consistent with an earlier study by Meyer (2008), who reported that older workers often exhibit a lower inclination to integrate novel techniques and technologies, including patient-handling devices, into their workplace practices. Fasbender et al. (2022) proposed two pathways that may explain this tendency: the motivational and capability pathways. The motivational pathway suggests that older workers, perceiving fewer future opportunities and less remaining time in their careers, may view new technology as less useful, leading to lower acceptance (Fasbender et al., 2022). The capability pathway highlights how older workers might perceive themselves as having slower processing speeds and diminished organizational abilities to assimilate new information, making new technologies seem more challenging to learn and implement (Fasbender et al., 2022). In addition to age, immigrant status was significantly associated with less use of coworker assistance and patient handling devices. This finding corroborates that of previous researchers (Lyu et al., 2018), who reported immigrant workers showed lower engagement in safe work practices due to several reasons, such as cultural differences, language barriers, and limited knowledge of available safety resources. However, given that our sample included only workers proficient in reading, writing, and understanding Korean, language barriers were unlikely to play a major role in this study. Instead, cultural norms and unfamiliarity with available safety resources may have been more influential. For instance, in many immigrant workers' home cultures, one-person patient handling is a common practice, and the use of patient-handling devices or coworker assistance may be rarely observed or encouraged.

Numerous researchers have documented the positive correlation between organizational safety climate and the implementation of various safety practices aimed at hazard mitigation and reducing injury rates among workers (Bosak et al., 2013; Engkvist, 2007; Lee & Lee, 2017). Within the broader concept of safety climate, management's safety priority has emerged as a crucial dimension (Teuma Custo et al., 2019). We have found that our results, specifically regarding the role of management's safety priority, are similar. Lee and Lee (2017) reported that organizational safety climate was an important factor for safe patient handling behaviors among nurses. Similarly, Engkvist (2007) studied attitudes toward a 'no lifting policy' program and highlighted the pivotal role of safety climate in fostering positive attitudes among nurses prior to the implementation of such policies. More specifically, Bosak et al. (2013) found a negative association between management's safety priority and workers’ risky behavior, such as ignoring safety rules. These researchers suggested that management's commitment to safety may promote worker's safety behavior.

We have found that the use of coworker assistance was associated with lower levels of overcommitment among workers. Overcommitment has been linked to diminished adherence to safe work behaviors. Prior researchers have supported this conclusion. Researchers studying critical care nurses found that overcommitment was negatively associated with safe work practices (Lee et al., 2010). Overcommitted workers often prioritize productivity and task completion over their safety. In their drive to meet deadlines or handle heavy workloads, they may perceive adherence to safe work behaviors, such as seeking coworker assistance, as time-consuming or inconvenient. This tendency can lead them to bypass proper safety protocols, increasing their risk of musculoskeletal injuries and other work-related health issues.

Job characteristics also play a role in shaping workers’ behaviors. Our findings suggest that workers with longer job tenures in direct care roles and those in non-permanent work arrangements were less likely to engage in coworker assistance. Contrary to our findings, Gyekye (2006) reported that workers with longer job tenures were more likely to commit to safe work behaviors (Gyekye, 2006). This discrepancy can be interpreted in several ways. Workers with more experience may have developed skills and greater confidence in their abilities to perform patient-handling tasks independently, not relying on or asking for help from colleagues. Therefore, they may have adapted both physically and psychologically to the demands of patient handling. Another possible explanation is the ‘healthy worker survivor effect,’ where those unable to manage patient handling independently may have left the job, leaving behind workers who can handle the tasks alone.

The association between non-permanent work arrangements and reduced coworker assistance is particularly noteworthy. As confirmed in our research, nearly three quarters of the participants were non-permanent workers, including those with temporary and independent work arrangements. This high proportion of non-permanent employment has significant implications for workplace dynamics and safety practices. Temporary employment has been shown to hinder social identification processes, leading to a lower sense of inclusion or belonging among workers (Lheureux & Parmentier, 2022). In South Korea, the situation is further complicated for independent workers, such as freelancers and self-employed individuals. According to the Labor Standards Act in South Korea, these workers are not classified as employees and, therefore, lack the legal entitlements afforded to standard employees (Ministry of Employment and Labor, n.d.). This legal distinction may exacerbate feelings of detachment from the workplace and its practices. These factors collectively contribute to a sense of disconnection among non-permanent workers, potentially making them perceive more barriers to engaging in collaborative safety practices, such as using patient handling devices and seeking coworker assistance.

### 4.3 Strengths and limitations

We have assessed the strengths of our study. First, we are the originators of research that highlights specifically the use of patient-handling devices and coworker assistance among direct care workers in South Korea. While it is well-documented that patient handling poses significant hazards to care workers’ musculoskeletal health and that mechanical lifting is the optimal way to prevent these symptoms, researchers in Korea have continued to focus on the negative effects of manual handling. To assess current practices in long-term care settings in Korea, we investigated the use of patient-handling devices, particularly in the context of their availability, and reliance on coworker assistance. By shedding light on these practices, we have provided valuable insights into the strategies direct care workers employ to manage patient-handling tasks and mitigate risks. These findings can inform workplace interventions, policy development, and future research aimed at improving the health and safety of direct care workers. Second, we collected a sample from 19 long-term care facilities in Gyeonggi Province in Korea, enhancing the generalizability of its findings. The inclusion of facilities with varying resource availability and workforce compositions provides a more comprehensive understanding of patient handling practices in real-world settings.

We have also noted a few limitations. First, we did not assess the frequency of using patient handling devices and coworker assistance. Even among workers who reported using both methods, there may be significant differences between those who utilized them regularly and those who used them only occasionally. This limitation may affect the accuracy of our findings by potentially underestimating the associations between these practices and musculoskeletal symptoms. Second, we employed a broader definition of patient-handling devices, encompassing mechanical lifts (overhead ceiling lifts, floor-based lifts), hoists, transfer belts and slide sheets, stand-assist devices, and repositioning aids. While this approach was necessary due to the low availability of advanced patient-handling devices in Korea, it may dilute the benefits of more sophisticated equipment. It could also reduce the sensitivity of multivariable analysis in identifying factors associated with advanced device use. Third, the cross-sectional design of the study limits the ability to demonstrate causal relationships between variables. Fourth, the reliance on self-reported measures introduces the possibility of reporting bias, influenced by memory lapses or social desirability bias, where participants may respond in ways they perceive as more socially acceptable rather than reflecting their actual experiences (Latkin et al., 2017). Finally, while the recruitment of participants from 19 long-term care facilities enhanced the diversity of the sample, the study sample may not be representative of direct care workers in all long-term care facilities in Korea. Specifically, the relatively small subgroup of immigrant workers may not fully capture the experiences of this population in different contexts. Differences in policies, resource allocations, and demographics of patients and nursing staff across countries may affect the applicability of our findings to international contexts.

## 5 Conclusion

We have highlighted significant differences in the prevalence, frequency, and severity of musculoskeletal symptoms among direct care workers based on their use of patient-handling devices and coworker assistance. Workers using patient-handling devices—whether alone or with coworker assistance—reported fewer and less severe symptoms, suggesting a potential protective effect. When manual handling was unavoidable, coworker assistance was associated with lower symptom severity compared to one-person manual handling. From the findings, we have revealed that workers who are overcommitted and have longer job tenure in long-term care, as well as marginalized workers (older workers, immigrants, non-permanent workers) and those perceiving low management's safety priority, were less likely to adopt patient-handling devices or coworker assistance. From the results, we emphasize the crucial role that nursing leaders may play in promoting safe patient handling behaviors of direct care workers, particularly among vulnerable worker groups. Future researchers should use larger, more diverse samples and include data on the type and frequency of patient handling device use and coworker assistance to enhance validity and generalizability. Longitudinal study designs would be particularly valuable in establishing causal relationships and tracking changes over time, addressing the limitations of cross-sectional studies.

## Funding

This research did not receive any specific grant from funding agencies in the public, commercial, or not-for-profit sectors.

## Institution and ethics approval

The study was approved by the Institutional Review Board of the University of California, San Francisco (Date: 05/20/2022, Identification number: #22-36334) and the Public Institutional Review Board in South Korea Date: 05/20/2022, Identification number:P01-202205-01-036).

## Disclaimer

None.

## CRediT authorship contribution statement

**Minjung Kyung:** Writing – review & editing, Writing – original draft, Resources, Methodology, Investigation, Conceptualization. **Soo-Jeong Lee:** Writing – review & editing. **Laura M. Wagner:** Writing – review & editing. **OiSaeng Hong:** Writing – review & editing.

## Declaration of competing interest

The authors declare that they have no known competing financial interests or personal relationships that could have appeared to influence the work reported in this paper.
