# Corrigendum to “Use of Patient Handling Devices and Coworker Assistance in Long-Term Care Settings: A Cross -Sectional Study” [International Journal of Nursing Studies Advances 8 (2025) 100317]

**DOI:** 10.1016/j.ijnsa.2025.100323

**Published:** 2025-04-30

**Authors:** Minjung Kyung, Soo-Jeong Lee, Laura M. Wagner, OiSaeng Hong

**Affiliations:** a College of Nursing, The Catholic University of Korea, Seoul, South Korea; b School of Nursing, University of California, San Francisco, San Francisco, California

The authors regret that the incorrect acknowledgement section was included in the article. The last sentence ‘The authors also thank research assistants (Bora Nam and Kevin Joiner) for their contribution to data collection’ in the acknowledgement section is incorrect.

The correct acknowledgement section is as follows:

